# Outcomes of Vitrectomy for Long-Duration Macular Hole

**DOI:** 10.3390/jcm9020444

**Published:** 2020-02-06

**Authors:** Xhevat Lumi, Mina Mahnic, Beáta Éva Petrovski, Goran Petrovski

**Affiliations:** 1 Eye Hospital, University Medical Centre Ljubljana, 1000 Ljubljana, Slovenia; mina.mahnic@gmail.com; 2 Faculty of Dentistry, University of Oslo, 0317 Oslo, Norway; beata.petrovski@odont.uio.no; 3 Center for Eye Research, Department of Ophthalmology, Oslo University Hospital and University of Oslo, 0450 Oslo, Norway; goran.petrovski@medisin.uio.no

**Keywords:** macular hole, visual acuity, macular hole diameter, ellipsoid zone

## Abstract

The present study investigated the functional and anatomical outcomes of idiopathic chronic macular hole (MH) surgery with different surgical approaches related to the chronicity and diameter of the MH. A comparative retrospective study between three groups of patients who underwent vitrectomy for long-duration MH (mean: 13.5 months) was conducted. In the first group of patients (G1 or IP), the internal limiting membrane (ILM) was systematically peeled; in the second group (G2 or IPEP), the ILM and epiretinal membrane (ERM) were peeled; and in the third group (G3 or IF), patients underwent inverted ILM flap technique surgery. Pre- and post-operative best corrected visual acuities (pre- and post-op BCVA) were studied. Macular optical coherence tomography (OCT) scans were performed to measure the MH minimum and maximum diameter pre-operatively, as well as to confirm its post-op closure and evaluate the integrity of the ellipsoid zone (EZ). Fifty eyes of 48 patients (33 female and 15 male) were retrospectively evaluated. MH closure rate was 100% in IP group, 66.7% in IPEP, and 95.2% in IF group. All three groups had a statistically significant improvement of BCVA. EZ post-op was restored in 88.2% of the cases from G1, 41.6% from G2, and 23.8% from G3. No statistically significant relationship between the smaller or larger MH diameter and the visual acuity improvement was found. Patients with chronic MH and ERM have worse functional and anatomical outcomes after surgery. Treatment of chronic MHs without ERM results in a better closure rate with either an inverted ILM flap approach or systematic ILM peel.

## 1. Introduction

Macular hole (MH) is a full thickness break in the central part of the neurosensory central fovea that causes poor vision and metamorphopsia [1,2]. It occurs most commonly in the 6th–7th decade of life and more often affects women than men [3,4]. There are different etiologies that can lead to MH; it can be idiopathic or secondary, and is caused by trauma, chronic cystoid macular edema, or vascular occlusion [3,5]. Gass and Johnson described the clinical staging and the evolution of idiopathic MH. Antero-posterior and tangential vitreous traction on the fovea can result in morphological changes that start with a macular cyst (stage 1 MH) and continue to a full thickness MH smaller than 400 µm (stage 2 MH). Further development results in stage 3 MH (greater than 400 µm in size and incomplete vitreous separation), and end with stage 4 MH, in which complete separation of the vitreous from the macula and the optic disk as well as frequent epiretinal membrane (ERM) development occurs [1,2,6]. 

Since its first description by Kelly and Wendell, the surgical management of MH by pars plana vitrectomy (PPV) and associated surgical techniques has been improved. Recent reports have shown an increased success rate of MH repair of greater than 90% [7,8,9]. Several reports of MH surgery have shown better functional and anatomical results in holes with shorter duration [10,11]. For holes stage 3 and 4 with pre-op latency of six months and more, an anatomical success rate with complete hole closure has been reported between 33% and 81% of the cases with a worse functional outcome [11,12,13].

Some authors have reported different criteria for classification of MH as chronic. The latter is assumed to be a hole that is present for more than six months [14]. For other authors, MH is chronic after one year from when the patient first noticed visual deterioration or from a documented ophthalmologic examination [15]. The outcomes of surgery are worse, depending on the duration of the hole and its diameter [10,16].

Different types of surgery for the treatment of chronic MH exist: vitrectomy (to obtain a complete posterior vitreous detachment (PVD)) [17], vitrectomy with peeling of the internal limiting membrane (ILM) [18], and vitrectomy with inverted ILM flap or temporal flap technique [19]. 

Recent reports have shown a less favorable surgical outcome in MH associated with epiretinal (atypical) tissue proliferation [20,21].

The purpose of this retrospective consecutive series of MH stage 3 and 4 with duration of more than six months is to report functional and anatomical surgery outcomes depending on the surgical approach, adjusted to hole distinctiveness.

## 2. Patients and Methods

Records of patients who underwent surgery for chronic full thickness macular hole (FTMH) stage 3 and 4 between 1 January 2014 and 30 April 2018 at Eye Hospital, University Medical Centre Ljubljana, Slovenia were evaluated. Patients from a single surgeon (XL) with idiopathic chronic FTMH and continuous complete post-op follow-up were included, while those with secondary MH were excluded. Since more complex mechanisms have been implicated in the development of myopic MH, such as strong adhesion of the vitreous cortex and complex vector forces exerted by posterior staphyloma, eyes with axial length (AL) greater than 25 mm were excluded [22].

Prior to surgery, all patients underwent a complete ophthalmological examination, including Snellen best-corrected visual acuity (BCVA), intraocular pressure measurement, slit lamp anterior segment examination, and dilated funduscopic examination. The Snellen acuity was converted into a logarithm of the minimum angle of resolution (logMAR) equivalent. Optical Coherence Tomography (Swept source OCT, DRI OCT Triton, Topcon) was performed and used for evaluation of the MH size pre-op; presence of epiretinal tissue around the hole, confirmation of the post-operative hole closure, and evaluation of the integrity of retinal layers including the ellipsoid zone (EZ) were also performed.

Included in the study were 50 eyes of 48 patients (33 female and 15 male). The duration of the MH was determined from the moment the patient first noticed significant visual loss or as documented on any available previous ophthalmologic examination report. 

On the OCT scans, the sizes of the smaller and larger diameter of the chronic FTMH were measured on the day before surgery. The minimum hole width was measured at the narrowest hole point in the mid retina, using the OCT caliper function, as a line drawn roughly parallel to the retinal pigment epithelium (RPE). The maximum hole width was measured at the largest hole point, above the RPE [5].

Patients were classified into three different groups according to the surgery that was performed. In the first group G1 (IP), the patients underwent PPV with systematic ILM peeling; in the second group G2 (IPEP), vitrectomy with an ERM and ILM peeling was performed; in the third group G3 (IF), vitrectomy with inverted flap technique was performed [17,18,19]. 

In all groups, the surgical technique involved a three-port PPV (23- or 25-gauge surgery). In cases with nuclear sclerosis or significant cataract, phacoemulsification with IOL in-the-bag implantation was carried out prior to and together with vitrectomy. In all patients, Briliant Peel Dye (FLUORON GmbH) was used for staining the ILM. Epiretinal tissue and ILM were removed using ILM forceps. ILM was removed in a circular fashion around the hole in 3-4-disc diameters. ILM flap was also performed with ILM forceps using a high magnification contact lens starting temporally to the hole. At the end of surgery, fluid/air exchange was performed with 10% of perfluoropropane gas as a tamponade (Alcon Laboratories, Fort Worth, TX). Patients were instructed to maintain face-down positioning up to one week. 

Crystalline lens status was evaluated in post-operative follow-up visits in all patients without previous cataract surgery. To avoid the impact of lens status in final BCVA, every patient with nuclear sclerosis had cataract surgery before final VA testing. 

Visual improvement was defined as an increase of BCVA on a Snellen VA chart. Anatomical success was defined as complete closure of the MH, determined by both ophthalmoscopic and OCT examination (only flat/closed) [23].

The analysis of the data was performed by descriptive statistical analysis; percentage distribution, mean and standard deviation (SD), median and interquartile range (IQR) are shown. The one-Way ANOVA with Bonferroni post hoc test was used to compare means of continuous, numerical variables. Homogeneity of variance was analyzed with Levene‘s test. Wilcoxon Signed Rank Test was used to compare two dependent variables, when the normality assumption was not satisfied. 

Chi-square (*χ*^2^) and Fisher’s exact test were used to check the differences of the distribution of categorical variables. Best-corrected visual acuity (BCVA) was recorded as a Snellen visual acuity and converted to logMAR for statistical analysis. Significance limit was set at *p* < 0.05. STATA (Stata version 14.0; College Station, TX, USA) were used for the statistical analyses.

The datasets analyzed during the current study are available by the corresponding author on request.

## 3. Results

Mean duration of MH before surgery was 13.5 months. The reported symptoms in most cases were blurred vision or image distortion; in three patients, a defect in the visual field was referred. 

In group IP, 17 eyes of 15 patients (nine females and six males) were included. The mean age was 68.8 ± 5.4 years (range: 61–76 years). The average size of the smaller hole diameter of the FTMH was 299.2 µm ± 151.6 (range: 45–572 µm), and the average size of the larger diameter was 806.0 µm ± 272.0 (range: 300–1513 µm). Two patients from this group had surgery for MH on both eyes (Table 1 and Table 2).

In group IPEP, 12 eyes of 12 patients (nine females and three males) were included. The mean age was 67.6 ± 7.0 years (range: 57–81 years). The average size of the smaller hole diameter of MH was 383.0 µm ± 149.4 (range: 226–732 µm), and the larger one was 903.2 µm ± 210.7 (range: 532–1281 µm) (Table 1 and Table 2). 

In group IF, 21 eyes of 21 patients (15 females and six males) were included. The mean age was 69.8 years ± 7.1 (range: 60–87 years). The average size of the smaller hole diameter was 458.1 µm ± 130.8 (range: 148–707 µm), and the larger diameter 950.6 µm ± 305.6 (range: 280–1543 µm) (Table 1 and Table 2). The gender and age showed no significant differences among the three groups. 

There were no intra- or post-op complications that required medical or surgical intervention. Mean post-op follow-up in IP group was 20.8 ± 6.0 months (median: 18; IQR: 16–23, range: 15–37 months), in IPEP group was 19.5 ± 3.8 months (median: 18; IQR: 18–19, range: 15–29 months), and in IF group was 16.9 ± 2.7 months (median: 16; IQR: 15–19, range: 14–22 months). At follow-up, all patients underwent complete ophthalmological examination and OCT. 

**Figure 1:**
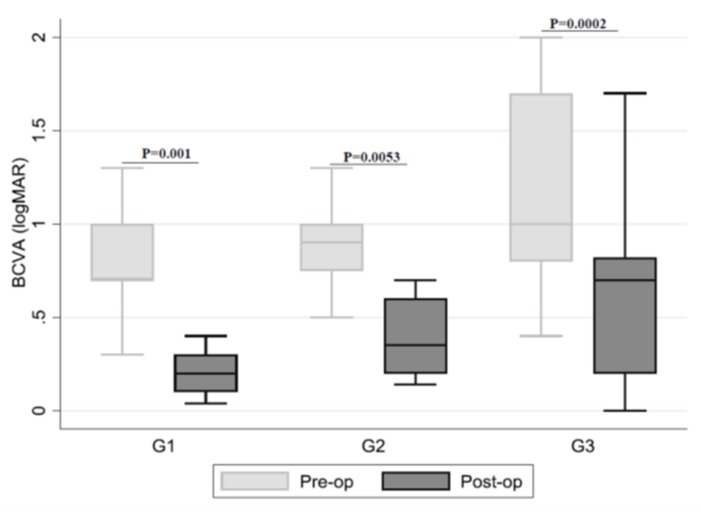
Relationship between pre- and post-op logMAR and groups (median, IQR, range). Legend: BCVA pre-op = best corrected visual acuity before surgery; BCVA post-op = best corrected visual acuity after surgery; logMAR = Logarithm of the Minimum Angle of Resolution; Statistical analysis was performed using the Wilcoxon Signed Rank Test.

The pre- and post-op BCVA in all groups is shown in Table 3 and Figure 1. The mean BCVA before surgery was 0.8 logMAR (range: 0.3–2.0) for group IP, 1.0 logMAR (range: 0.5–2.0) for group IPEP, and 1.2 logMAR (range: 0.4–2.0) for group IF. Statistically significant improvement was apparent in all groups, while significant association between anatomic MH closure and surgery type/group (*p* = 0.007) (the proportion of anatomic MH closure) was the highest in group IP (100%, *p* = 0.001) and in IF (95.2%, *p* = 0.0002).

**Figure 2:**
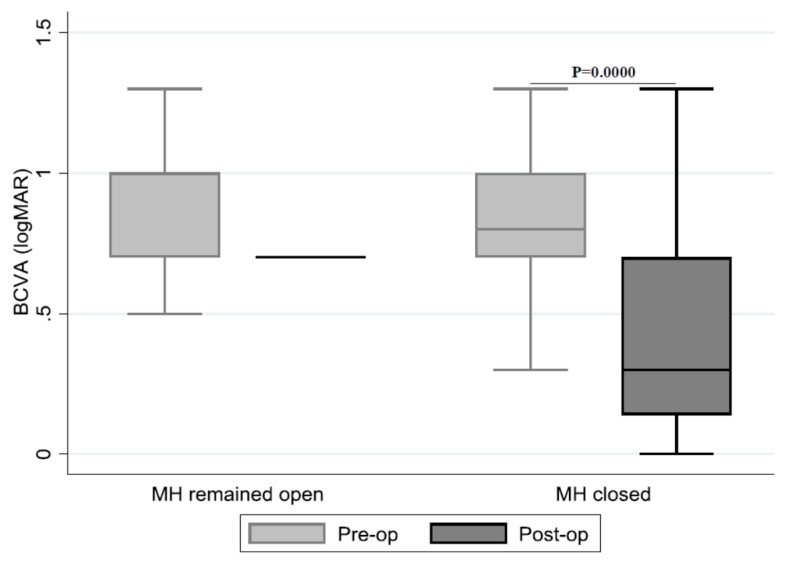
Relationship between pre- and post-op logMAR and closure of the macular hole (median, IQR, range). Statistical analysis was performed using the Wilcoxon Signed Rank Test.

The improvement in visual acuity with the frequencies and percentages of distribution of patients, as well as the relationship between visual acuity and closure of MH, is shown in Table 4 and Figure 2. No significant proportion differences could be detected between the improvement in VA and the duration of the symptoms and closure status of the MH. 

No statistically significant relationship between the smaller or larger MH diameter measurement and the visual acuity improvement was found (*p* = 0.778 and 0.102, respectively) (Table 5).

**Figure 3:**
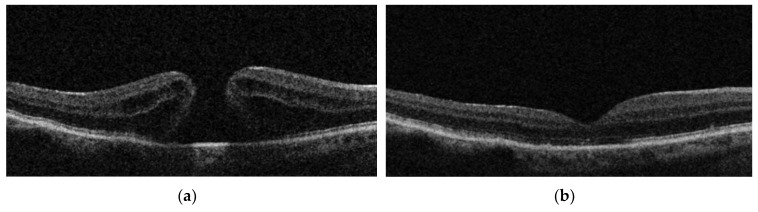

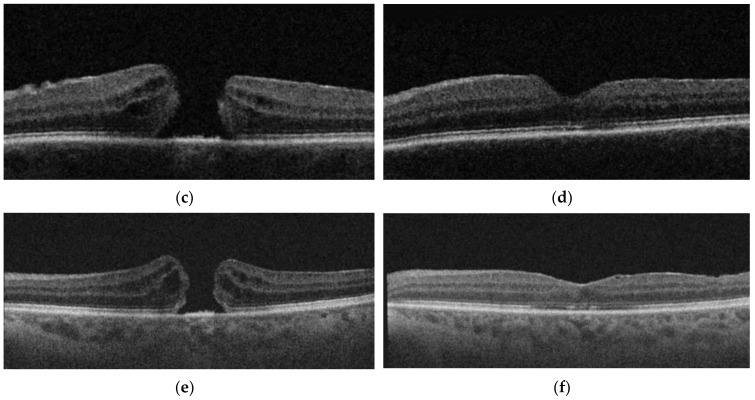
Representative OCT finding pre- and post-operatively for each of the three groups studied. IP group: (**a**) = pre-op OCT, and (**b**) = post-op OCT after ILM peeling; IPEP group: (**c**) = pre-op OCT, and (**d**) = post-op OCT after ERM + ILM peeling; IF group: (**e**) = pre-op OCT, and (**f**) = post-op OCT after inverted flap technique.

A representative OCT finding pre- and post-op for each of the three groups studied is shown in Figure 3.

In group IP, 100% of the eyes reached anatomic closure of the MH; in group IPEP, 66.7% (8/12), and in IF the closure rate was 95.2% (20/21), assessed both by a clinical and OCT examination.

The EZ returned to its integrity in the 88.2% of patients in IP group; in 41.6% in IPEP, and in 23.8% of patients in group IF (Table 6). The integrity of the EZ or the photoreceptors, and the MH closure, showed nearly a significant relationship (*p* = 0.05), while the RPE integrity was present in all cases studied; thus, it could not be correlated with the MH closure. Furthermore, a significant relationship was found between the MH closure and the surgery type/groups (*p* = 0.012).

In group IP, at the final examination, one patient had an irregularity of the inner retinal layers; one case had an ERM, and another had intraretinal edema with hyperreflective dots. In group IPEP, one patient had subretinal fluid at the final examination. In group IF, one patient had ERM, while another had irregularity of the inner retinal layers, and a third patient had some small intraretinal pseudocysts (Table 6).

## 4. Discussion

Currently, the most standard procedure for treating MH is PPV with peeling of the ILM and intraocular tamponade followed by face-down positioning for 3–7 days. In different studies, it has been demonstrated that vitrectomy with ILM peeling gives a better hole closure ratio [8,24,25].

Cytologic analysis performed by Yooh et al. has shown that ILM around MHs contains myofibrocytes [26]. It has been suggested that contraction of these myofibrocytes can cause enlargement of the MH and prevent its closure [26]. Our group has shown the presence of progenitor cells in ILMs [27], and ILM may act as a scaffold for the proliferation of cellular components that can result in tangential traction around the fovea, causing reopening of the MH [8,25,28]. The ILM peeling might also stimulate gliosis by effecting trauma locally around the MH and helping to close the hole [28,29].

In one study that used spectral domain OCT for the evaluation of the pathological changes of the retinal layers, it was demonstrated that these layers after MH surgery become restored first at the external limiting membrane, followed by the EZ and then by the cone outer segment [30]. With the evaluation of these structures, one can evaluate the photoreceptors’ state, which in turn is a predictive sign of morphological and functional recovery of photoreceptors [30,31]. The reconstruction of the EZ can thus be considered to be a good prognostic factor for visual rehabilitation after MH surgery [31]. 

In a retrospective study that investigated the morphologic characteristics of chronic MH using OCT, it was found that the minimum hole diameter was significantly larger in chronic MH than in acute ones [15]. Our study showed no such relationship between smaller or larger MH diameter and visual acuity improvement. The larger minimum hole diameter observed in chronic MH patients may be otherwise correlated with the poorer visual prognosis in these types of holes. Photoreceptors may be displaced in these MH and changed over time with atrophy or necrosis [32,33,34,35].

Most studies demonstrate clear benefits if surgery is performed within six months of the onset of symptoms, and suggest an inverse relationship between the duration of symptoms of FTMH and surgical success in terms of closure and visual acuity improvement [12,17].

Although the small number of cases in our study may be a limitation in itself, the consistent use of the three different types of surgery undertaken for the treatment of chronic MHs showed the final BCVA is dependent on the MH closure and the pre-op visual acuity. All groups reached a statistically significant improvement of the visual acuity, out of which more than half achieved >0.4 improvement on logMAR or equivalent to four lines or 20 letters. The group where the ERM was present at the time of surgery (IPEP), and had a worse hole closure ratio than the ILM peeling or the inverted ILM flap technique; furthermore, there appeared to be a significant relationship between MH closure and surgery type.

ERM associated with idiopathic MH has been described as atypical retinal tissue or epiretinal proliferation [20,21]. Immunohistochemical analysis of the specimens of this tissue has shown this to be a gliotic or fibrotic process as a consequence of retinal glial and transdifferentiated Müller cells’ reaction [36]. The presence of the ERM is due to a chronic pathogenic process associated with more severe damage to EZ [20], which, according to some reports, is associated with worse functional and anatomical outcome of MH surgery [20]. In our group of patients with epiretinal tissue around the hole (IPEP group), worse anatomical results of surgery can be related to the impact of such changes.

A very good anatomical restoration of the EZ in IP group (88.2%) was achieved, which was worse in the other two groups. It can be speculated that the worse post-op result regarding the integrity of the EZ in group IPEP was due to the presence of ERM and wider minimum and maximum diameter of the holes. In group IF, the worse restoration of the EZ can also be assumed to be due to presence of a wider diameters of the MH pre-operatively. The RPE maintained its integrity in all patients of all groups.

Finally, OCT scans showed changes like irregularity of the inner retinal layers, ERM, subretinal fluid, or intraretinal pseudocysts, in a few patients of all groups, but that appeared not to cause any significant visual deterioration.

A limitation of the study is the small sample size; however, if a large difference existed between two groups, such a difference would be detectable even in a smaller sample size. A larger sample size and/or multicenter study would thus be needed to further validate the findings in the present analysis.

## 5. Conclusions

This study shows that patients with chronic MH and the main duration of symptoms of 13.5 months can gain both anatomical and functional success from ILM peeling or inverted ILM flap technique surgery, while the presence of ERM with MH has a worse hole closure rate. 

## Figures and Tables

**Table 1:** Characteristics of the studied population.

Groups	Eyes *N* = 50 (%)	Patients *N* = 48 (%)	Female Patients *N* = 33 (%)	Male Patients *N* = 15 (%)	Age Mean ± SD Range (Years)
**IP**	17 (34.0)	15 (31.2)	9 (27.3)	6 (40.0)	68.8 ± 5.4 61–76
**IPEP**	12 (24.0)	12 (25.0)	9 (27.3)	3 (20.0)	67.6 ± 7.0 57–81
**IF**	21 (42.0)	21 (43.8)	15 (45.4)	6 (40.0)	69.8 ± 7.1 60–87

Groups: IP = internal limiting membrane (ILM) peeling; IPEP = ILM and epiretinal membrane (ERM) peeling; IF = inverted ILM flap technique; and N = number. Statistical analysis was performed using the One-Way ANOVA and *χ*^2^ tests.

**Table 2:** Optical Coherence Tomography (OCT) characteristics of full thickness macular hole (FTMH).

Groups	Smaller Diameter MH (µm) Mean ± SD (Range)	Larger Diameter MH (µm) Mean ± SD (Range)
**IP**	299.2 ± 151.6 (45–572)	806.0 ± 272.0 (300–1513)
**IPEP**	383.0 ± 149.4 (226–732)	903.2 ± 210.7 (532–1281)
**IF**	458.1 ± 130.8 (148–707)	950.6 ± 305.6 (280–1543)

Statistically significant differences occurred in the smaller diameter macular hole (MH) between groups IP and IF (*p* = 0.004); 1 outlier (2828 µm) in the large diameter measurements of the MH in group IF was removed from the data analysis. Statistical analysis was performed using the One-Way ANOVA with Bonferroni post hoc test.

**Table 3:** Functional and anatomical results with comparison of the visual acuity before and after surgery.

Groups	BCVA Pre-op Logarithm of the Minimum Angle of Resolution (logMAR) Mean ± SD, Median (IQR), Range	BCVA Post-op (logMAR) Mean ± SD, Median (IQR), Range	Anatomic MH Closure N (%)	*p*-Value
**IP**	0.8 ± 0.4 0.7 (0.7–1.0) 0.3–2.0	0.3 ± 0.3 0.2 (0.1–0.3) 0.4–1.0	17/17 (100.0%)	0.001
**IPEP**	1.0 ± 0.4 0.9 (0.7–1.0) 0.5–2.0	0.4 ± 0.3 0.4 (0.2–0.6) 0.1–1.3	8/12 (66.7%)	0.0053
**IF**	1.2 ± 0.5 1.0 (0.8–1.7) 0.4–2.0	0.6 ± 0.5 0.7 (0.2–0.8) 0.0–1.7	20/21 (95.2%)	0.0002

Legend: BCVA pre-op = best corrected visual acuity before surgery; BCVA post-op = best corrected visual acuity after surgery; MH = macular hole; and N = number. Statistical analysis was performed using the Wilcoxon Signed Rank Test and the *χ*^2^-test.

**Table 4:** Distribution of patients according to the improvement in visual acuity and duration of the symptoms and closure status of the MH.

(Pre-op)–(Post-op) logMAR Difference	(%)	MH Remained Open (Frequency (%))	MH Closed (Frequency (%))
<0	3 (6.0)	1 (20.0)	2 (4.4)
0–0.2	10 (20.0)	2 (40.0)	8 (17.8)
0.2–0.4	9 (18.0)	1 (20.0)	8 (17.8)
>0.4	28 (56.0)	1 (20.0)	27 (60.0)

Legend: BCVA Pre-op = best corrected visual acuity before surgery; BCVA Post-op = best corrected visual acuity after surgery; and MH = macular hole. Statistical analysis was performed using the Fisher’s exact test.

**Table 5:** Relationship between difference MH diameters and change or improvement in visual acuity.

	**(Pre-op)–(Post-op) logMAR Difference (*n*)**			
**Smaller MH** **Diameter (µm)**	**<0** ***N*** **(%)**	**0–0.2** ***N*** **(%)**	**0.2–0.4** ***N*** **(%)**	**>0.4** ***N*** **(%)**
**0–250**	1 (33.3)	2 (20.0)	2 (22.2)	6 (21.4)
**250–400**	0 (0.0)	2 (20.0)	4 (44.4)	10 (35.7)
**>400**	2 (66.7)	6 (60.0)	3 (33.3)	12 (42.9)
	(**Pre-op)–(Post-op) logMAR Difference (*n*)**			
**Large MH** **Diameter (µm)**	**<0**	**0–0.2**	**0.2–0.4**	**>0.4**
**0–250**	0 (0.0)	0 (0.0)	0 (0.0)	0 (0.0)
**250–400**	0 (0.0)	1 (16.7)	2 (33.3)	0 (0.0)
**>400**	2 (100.0)	5 (83.3)	4 (66.7)	17 (100.0)

Legend: BCVA pre-op = best corrected visual acuity before surgery; BCVA post-op = best corrected visual acuity after surgery; MH = macular hole, µm: micrometer, and N: number. Statistical analysis was performed using the Fisher’s exact test.

**Table 6:** Post-op anatomical results.

Groups	Integrity of Ellipsoid Zone (%) *N*	Integrity of RPE (%)	Characteristics Post-op
**IP**	88.2% (15/17)	100%	5.9% (1/17) Irregularity of inner retinal layers 5.9% (1/17) ERM 5.9% (1/17) Intraretinal edema + hyperreflective dots
**IPEP**	41.6% (5/12)	100%	8.3% (1/12) Subretinal fluid
**IF**	23.8% (5/21)	100%	4.8% (1/21) Irregularity inner retinal layers 4.8% (1/21) ERM 4.8% (1/21) Intraretinal pseudocysts

Legend: RPE = retinal pigment epithelium; N = number. Statistical analysis was performed using the *χ*^2^-test.

## Abbreviations

MH	macular hole
FTMH	full thickness macular hole
ERM	epiretinal membrane
PPV	pars plana vitrectomy
ILM	internal limiting membrane
AL	axial length
BCVA	best corrected visual acuity
VA	visual acuity
OCT	optical coherence tomography
EZ	ellipsoid zone
RPE	retinal pigment epithelium
logMAR	the Logarithm of the Minimum Angle of Resolution
IP	group of patients that underwent PPV with systematic ILM peeling
IPEP	group of patients that underwent vitrectomy with an ERM and ILM peeling
IF	group of patients that underwent vitrectomy with inverted flap technique
